# The mediating effect of hope agency on perceived stress and professional burnout among Polish corporate employees

**DOI:** 10.1038/s41598-024-52289-9

**Published:** 2024-01-22

**Authors:** Małgorzata Szcześniak, Adam Falewicz, Marcin Wnuk, Grażyna Bielecka, Daria Madej

**Affiliations:** 1 Institute of Psychology, University of Szczecin, Szczecin, Poland; 2 Department of Work and Organizational Psychology, Faculty of Psychology and Cognitive Sciences, Adam Mickiewicz University, Poznań, Poland

**Keywords:** Human behaviour, Quality of life

## Abstract

Job burnout is considered an outcome of prolonged exposure of employees to stress. Although many studies have focused on the presence of a direct association between stress and burnout, we still know very little about mediators that indirectly play a role in this relationship. Previous analyses have determined that self-efficacy acts as a mechanism that explains the overall relationship between stress and burnout. However, there is no such evidence to support the mediatory function of hope. Therefore, the main aim of the current study was to verify whether self-efficacy, hope pathways, and hope agency are mediators in this relationship. The study included 408 Polish-speaking adults who completed the Perceived Stress Scale, the Maslach Burnout Inventory, the Generalized Self-Efficacy Scale, and the Dispositional Hope Scale. The outcomes indicated a positive correlation of stress with the overall burnout score, as well as all subscales. Moreover, hope agency was a mediator, thus suggesting that there is also an indirect relationship between stress and job burnout. Therefore, it can be assumed that higher stress is associated with lower motivation to generate and sustain the actions needed to reach the goals. Consequently, lower hope agency may lead stressed employees to greater exhaustion and reduced personal accomplishment.

## Introduction

Work plays an important role in human life^1^. Engagement in decent work provides a means of survival^2^, gives employees an opportunity for personal growth^3^, satisfies the need of connection with others^4^, fosters mental health^5^, and sustains psychological functioning^3^. At the same time, working may have adverse consequences on people's lives^6^ and inhibit their well-being^2^.

According to OECD 2021 data, Poland ranks high among the countries covered by the study in terms of working time, exceeding 1800 h for worker per year. Even though in the last quarter of 2022, 74% of Polish employees declared being satisfied with their present job^7^, there is some evidence that others are exposed to high levels of stress and occupational burnout^8^.

### Stress and job burnout

Job burnout is considered an unpleasant condition^9^ and individuals' response to their tiredness due to work. According to one of the most recognized conceptual models of burnout, this specific type of work-related phenomenon^10^ consists of three key components: (1) overwhelming exhaustion that reflects increased strain and feelings of being depleted of emotional vitality^11^; (2) feelings of cynicism (or depersonalization) that are characterized by emotional and cognitive distancing from one's work, clients or people one works with; (3) the sense of ineffectiveness that refers to expressed in lower levels of efficiency, coping, and morale^12,13^.

There is conceptual evidence that burnout results from prolonged exposure to stress^12,14^ understood as a process by which specific situational conditions are assessed by the workers as exceeding their own resources^15^. Empirical results show that work-based stress is one of the most important burnout antecedents^16,17^, explaining approximately 30% or more of its variance^18,19^. People who experience persistent occupational stress tend to respond with higher levels of weariness caused by their job (overwhelming exhaustion), a detached attitude toward the job or co-workers (emotional and cognitive distancing), and negative emotions or cognitions about their own accomplishments (the sense of ineffectiveness)^20^.

### Self-efficacy and hope: potential mediating mechanisms

Although many studies have confirmed the existence of a direct association between stress and burnout among various professional groups or athletes, there is still little knowledge about potential mediators that indirectly play a role in this relationship. Previous analyses have determined that psychological capital^21^, sleep quality^22^, and self-efficacy^23,24^ act as mechanisms that explain the overall relationship between stress and burnout. Unlike environmental factors or working conditions that are difficult to change, cognitions (e.g., self-esteem) and motivations (e.g., hope) can be modified^20^. Since both self-efficacy and hope are seen as psychological resources that have a large impact on occupational burnout^25^, we have attempted to verify their mediating role in the relationship between perceived stress and job burnout.

Self-efficacy is usually conceptualized as the belief in one's ability to cope with different challenging demands^26,27^. Its buffering potential against stress and burnout^23,24^ stems from the fact that self-efficacy is a personal factor that allows one to cope with adversity^28^ thus reducing the risk of burnout^29^. In fact, Maslach^30^ has noted that the evaluation of oneself is one of the most significant variables for burnout. Other studies have found that people facing high levels of pressure in their work and having problems in dealing with them are more inclined to develop lower self-efficacy and be tired of working. Such relationships were noted, for example, in groups of Chinese teachers^24^ and Polish air traffic controllers^23^.

Hope is a construct that relates to individual differences in having goals and the means to reach them^31^. According to one well-known theory of hope, hopeful people are those who display goal-oriented thoughts (hope pathways) and are motivated to pursue them (hope agency). Several researchers have seen hope as a psychological resource that is associated with the sense of uncertainty arising in threatening situations^32^. Moreover, hope has been recognized as an important buffer against exhaustion^33^. Ho and Lo^34^ have found that hope accounted for 38.4% of the variance in job burnout and continued to be its significant predictor even after controlling for confounders.

Although there are no studies on the mediating role of hope in the relationship between stress and occupational burnout, there is empirical evidence to suggest that such an effect is possible. For example, Liu et al.^35^ have provided support for the theory that hope is a mediator between peer victimization and emotional difficulties. By analogy, it can be assumed that employees experiencing stressful life events may feel unable not only to generate routes toward a desired goal (hope pathways) but also to initiate goal pursuit (hope agency), which may lead to the development of exhaustion, depersonalization, and reduced personal accomplishment.

### Present study

Based on the evidence described above, we hypothesized that perceived stress is positively associated with job burnout (Hypothesis 1). Moreover, we expected that both stress and burnout would correlate negatively with self-efficacy and dispositional hope (Hypothesis 2) in its two dimensions (hope pathways and hope agency). In addition, we assumed the buffering hypothesis of self-efficacy, hope pathways and hope agency according to which these three variables act as parallel mediators in the relationship between stress and job burnout (Hypothesis 3), leading to a decrease in their strength in the presence of the three mediators.

## Method

For reporting this study, the Strengthening of Reporting of Observational Studies in Epidemiology (STROBE) checklist was utilized.

### Study design and participants

In the present study, a cross-sectional survey was conducted using convenience sampling. The selection for the research group was purposeful and targeted people working professionally. The research protocol was approved by the Bioethics Committee of the Institute of Psychology at the University of Szczecin (No. KB 5/2022) and the study was carried out consistent with the Declaration of Helsinki. The participants gave their written informed consent.

### Setting

For the purpose of this study, it was planned to recruit respondents using social media (mainly via Facebook and Instagram) and subsequent respondents were obtained using the snowball method, with corporate employment as the main criterion for inclusion. Data was acquired using an online platform.

### Variables

The dependent variable was the level of professional burnout. Perceived stress was taken as a predictor, while the mediating variables were self-efficacy and level of hope, understood as both agency and pathways. We also considered sex, age, the fact of working or not working in one's profession, the number of jobs, and years worked as potential confounders.

### Measurement

#### Perceived Stress Scale

We applied the Perceived Stress Scale^36^ to measure global perceived stress. This scale consists of 10 items, 4 of which are positively worded and the other six negatively. Respondents assess their level of agreement to each of the 10 statements by using a 5-point Likert scale ranging from 1 = never to 5 = very often. The total score can range from 0 (no stress) to 40 (high stress). The Cronbach’s alpha of the original English version of the questionnaire was from 0.74 to 0.91 and in the current study, the α was 0.89.

#### Maslach Burnout Inventory

To assess burnout, we used the Maslach Burnout Inventory^37^. The MBI contains 22 items and encompasses 3 subscales: emotional exhaustion, depersonalization, and personal accomplishment. Participants rate all the statements on a 7-point scale (from 0 = never to 6 = daily). The results are calculated separately for each subscale and for the overall score. A high level of burnout is evidenced by high results on emotional exhaustion and depersonalization, and low scores on personal accomplishment. The Cronbach's α coefficient for the whole scale was 0.89.

#### Generalized Self-Efficacy Scale

The Generalized Self-Efficacy Scale^26^ is a short, 10-item scale to assess the general sense of perceived self-efficacy, which reflects an optimistic belief in coping with everyday adversities. Respondents rate their level of agreement with each of the 10 statements on a 4-point Likert scale (1 = not true at all, 4 = exactly true). The total score is computed by summing all items. A higher score indicates higher self-efficacy. In the current study, the reliability coefficient of the GSES was α = 0.88.

#### Dispositional Hope Scale

We employed the Dispositional Hope Scale^38^ to assess hope through tapping pathways (ability to create options for reaching goals; α = 0.84) and tapping agency (motivational determination in using pathways to reach desirable goals; α = 0.83). The scale consists of 12 statements that respondents assess on an 8-point Likert scale (from 1 = definitely not true to 8 = definitely true). The questionnaire assesses two dimensions of hope and its overall score (α = 0.89).

### Study size

To determine the optimum sample size in advance, an a priori power analysis was performed, using G*Power 3.1.9.4 with a bivariate normal model correlation^39^. A small effect size of 0.17 with alpha of 0.05 and a power of 0.95 were set. The analysis showed that a total sample size would demand at least 366 participants. The rationale for using the value of Pearson’s *r* = 0.17 was based on meta-analyses of social psychology effects performed by Richard, Bond, and Stokes-Zoota^40^ who indicated mean $\overline{r}$ = 0.17 for the studies of health psychology.

### Statistical methods

All the statistics were conducted using the SPSS statistical package version 20. There was no problem with missing data. The normality of the distribution was checked for all variables, applying skewness and kurtosis not larger than ± 2. Ratner’s^41^ classification was used to assess the strength of Pearson's correlations, which indicates a weak correlation as lower than 0.3, moderate—between 0.3 and 0.7, and strong—more than 0.7. To detect multicollinearity, the Variance Inflation Factor (VIF) was examined (cut-off point = 5) and a tolerance value less than 0.1 was assumed.

To verify the presence of potential outliers, the Mahalanobis (*p* < 0.001) and Cook’s distance (< 1) were measured. The respondents’ sex, age, the fact of working or not in their profession, the number of jobs, and years worked in the profession were inserted as prospective confounders in Step 1. We examined them as potential confounding variables since some of them were found to influence both the predictors and professional burnout, thus, suggesting a spurious relationship.

**Figure 1:**
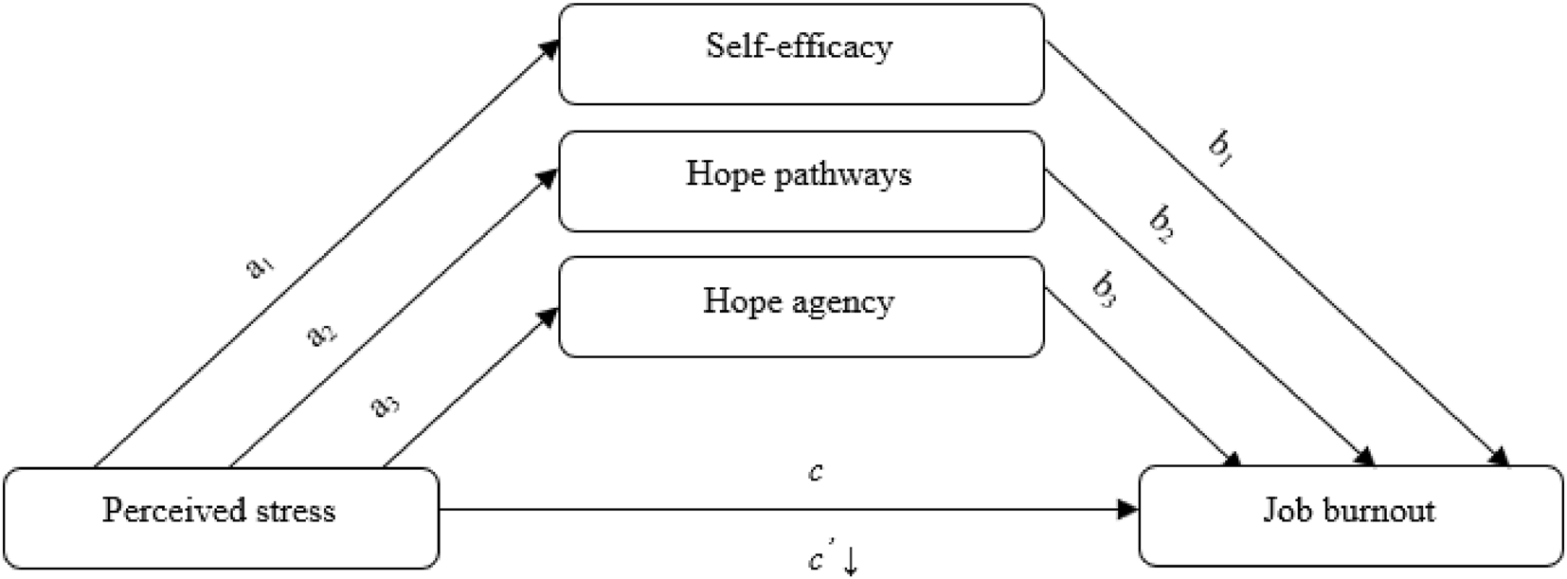
Hypothesized parallel model of the role of self-efficacy, hope pathways, and hope agency in the relationship between perceived stress and job burnout.

To test the hypothesis regarding the mediating effect of self-efficacy, hope pathways, and hope agency, a multiple mediation model with three parallel mediators (Model no. 4) was examined with the use of the PROCESS macro (version 3.2)^42^. Perceived stress (PS) was the independent variable, and job burnout (JB) was the dependent variable. Generalized self-efficacy (GSE), hope pathways (HP), and hope agency (HA) were included as mediators. Thus, three pathways were proposed: PS → GSE → JB; PS → HP → JB; and PS → HA → JB. For a statistical test of the parallel mediation, we followed the rule showing that all variables should be related with each other^43^. We used 5000 bootstrap samples and 95% confidence intervals to calculate the indirect effects. The hypothesized parallel model is presented in Fig. 1.

## Results

### Descriptive and correlational findings

The participants were 408 Polish-speaking adults (79% women and 21% men; age range: 21–57 years, *M* = 30.88, *SD* = 6.51). The prevalence of women in the study resulted from the fact that the dissemination of the link took place in specific departments of the companies in which women prevail (e.g., accounts, human resources). Moreover, women usually express greater willingness to take part in research confirmed in several studies^44^. This limits the generalizability of the study to the male population.

Regarding working in their field of expertise, 52% gave a positive answer and the remaining participants answered negatively. For 12% of the participants, the job was their first one, while for the remaining 88%, it was their second or third job. To the question regarding the number of years worked in the profession, the respondents answered as follows: 41% had worked less than 5 years, 20%—between 5 and 10 years, 12%—between 10 and 15 years, 4%—between 15 and 20 years, 3%—more than 20 years, and 20%—did not work in their profession at all.

Preliminary analyses showed that none of the variables considered (perceived stress, job burnout, perceived self-efficacy, and dispositional hope) exceeded the value of ± 2, suggesting their normal distribution (Table 1).

**Table 1:** Mean (*M*), standard deviation (*SD*), skewness, kurtosis, and correlations of the study variables (N = 408).

	*M*	*SD*	Skewness	Kurtosis	1	2	3	4	5
1. Stress	28.92	7.28	− 0.07	− 0.53	1				
2. Burnout	48.35	11.42	0.15	− 0.53	0.68***	1			
3. Self-efficacy	29.22	4.66	0.04	0.05	− 0.56***	− 0.49***	1		
4. Hope path	24.63	4.61	− 0.61	0.51	− 0.42***	− 0.42***	0.65***	1	
5. Hope agency	22.46	5.39	− 0.62	0.21	− 0.56***	− 0.55***	0.68***	0.70***	1

Note: *** p < 0.001.

All correlations confirmed Hypothesis 1 and Hypothesis 2. Stress and job burnout were moderately negatively associated with self-efficacy, hope path, and hope agency. The relationship between stress and job burnout was positive and moderate.

The VIF values ranged between 1.028 and 2.501, and the lowest tolerance was 0.400, indicating a low likeliness of multicollinearity. The Mahalanobis distance showed a chi-squared value of less than 0.001 in three of 408 cases. However, the statistics with and without the presence of the outliers were very similar, therefore, we decided not to remove them from the dataset as they were not influential. Moreover, the Cook's distance values (between 0.000 and 0.032) confirmed that the outliers were not problematic.

The linear regression showed that none of the variables included in Step 1 had a confounding effect. Indeed, they explained only 1.8% of the variance (*R*^*2*^ = 0.018): sex (β =  − 0.034, *t* =  − 0.817, *p* = 0.415), age (β =  − 0.111, *t* =  − 1.952, *p* = 0.052), the fact of working or not in their profession (β = 0.014, *t* = 0.317, *p* = 0.751), number of jobs (β = 0.005, *t* = 0.131, *p* = 0.895), number of years worked in their profession (β = 0.076, *t* = 1.267, *p* = 0.206). Self-efficacy (β =  − 0.226, *t* =  − 3.790, *p* = 0.001), hope pathways (β = 0.006, *t* = 0.100, *p* = 0.921), and hope agency (β =  − 0.397, *t* =  − 6.155, *p* = 0.001) predicted a significant amount of the variance (additional 31.8%) even after controlling for the effects of potentially confounding variables.

### Mediating role of hope agency

Hypothesis 3 was partially confirmed. The outcomes from a parallel mediation (Table 2) indicate a significant indirect effect of stress on job burnout through hope agency (PS → HA → JB). In fact, the total effect (weight c; (1.0634; B(SE) = 0.0572; 95%CI [0.9509; 1.1758]) was reduced to direct effect (weight c′; (0.8312; B(SE) = 0.0698; 95%CI [0.6940; 0.9684]) as stress had indirect impact on job burnout through hope agency. The parallel mediation model was significant with *R*^*2*^ = 0.50, *F*(4, 403) = 102.15, *p* = 0.001. In addition to this, the results also show a non statistically significant indirect effect of stress on job burnout via generalized self-efficacy (PS → GSE → JB) and hope pathways (PS → HP → JB) as in both cases the confidence intervals contain zero.

**Table 2:** Role of the role of self-efficacy, hope pathways, and hope agency in the relationship between perceived stress and job burnout (N = 408).

	a1, a2, a3 paths	b1, b2, b3 paths	Indirect Effect	B(SE)	Lower CI	Upper CI
PS → GSE → JB	− 0.36***	− 0.07(ni)	0.2322	0.0437	− 0.0606	0.1223
PS → HP → JB	− 0.26***	− 0.08(ni)	0.0228	0.0337	− 0.0456	0.0855
PS → HA → JB	− 0.41***	− 0.44***	0.1837	0.0459	0.0966	0.2775

Note: *** p < 0.001.

ns, not significant; PS, perceived stress; GSE, generelized self-efficacy; JB, job burnout; HP, hope pathways; HA, hope agency.

## Discussion

The goal of the current study was to demonstrate the relationship between stress and job burnout (Hypothesis 1), verify the associations between both stress and burnout, generalized self-efficacy, hope pathways, and hope agency (Hypothesis 2), and prove the buffering role of self-efficacy and both dimensions of hope in this relationship (Hypothesis 3). The results indicated a positive, moderate correlation of perceived stress of employees hired in the corporate work environment with the overall burnout score, as well as all subscales. It suggests that perceived life stress is associated with the risk of experiencing a sense of emotional exhaustion, a sense of separation from those around them, cynicism, and a sense that the person is accomplishing little.

This outcome is in line with research by Shoji et al.^20^, who provided evidence of the impact of chronic stress in the workplace on the level of burnout. Multiple studies show that, regardless of the profession, stress at work is positively linked to burnout^45–48^. Our research sheds new light on this relationship since refers to the general level of stress (not specifically connected to work; the same approach was adopted by Yu et al.^24^) and controls for possible confounders (gender, age, job in profession, number of previous jobs, number of years worked).

Moreover, stress shows a negative relationship with the presence of generalized self-efficacy and both dimensions of hope. It is theoretically well founded, especially in social cognitive theory, where self-efficacy determines consequences that are related to stress, such as burnout^49^. Experienced stress leads to a person's reduced belief in their competence to handle a wider scope of challenging situations. Conversely, lowered self-efficacy “opens” a path to burnout in the workplace^50^. The protective role of self-efficacy, understood as the increase of skills countering stress, was confirmed in numerous studies^51–53^. Research on interventions elevating levels of self-efficacy and its buffering effects against stress and burnout have yielded promising results^54–59^. Likewise, the presence of hope, understood as a positive emotional state that supports working people in achieving their goals, is negatively associated with the level of job burnout^56^.

Hope agency was a mediator, thus indicating that, beyond the direct association, there is also an indirect relationship between stress and job burnout. It can be assumed that people who report higher stress levels are more likely to present lower perception of how able they are to generate and sustain the actions needed to reach their goals (hope agency). They may have less motivation to pursue their goals because of too elevated stress and, consequently, display some typical signs of burnout, experience higher exhaustion, become more emotionally and cognitively distant from their work, and show reduced personal accomplishment. In fact, other studies suggest that engagement in repetitive self-referential thoughts that have a mere function of emotional regulation often offer ideas of inadequacy^57^, unrealistic fantasies, or an altered perception of the self^58^. Thus, in the long run, a less adaptive rumination can have the possible maladaptive implications because it fosters the sense of failure^59^, reduces perceived self-efficacy^60^, and increases burnout^61^. On the other hand, people who present lower levels of stress may manifest higher hope agency and thus experience lower burnout. Therefore, potential interventions enhancing the initial levels of motivational hope agency can minimize the influence of stress and burnout outcomes.

In contrast to other studies, self-efficacy was not a mediator between stress and burnout. Although this outcome seems surprising in the context of previous research^62,63^, it may suggest that hope agency better explains the relationship between perceived stress and job burnout. In terms of practical implications, this may draw attention to strengthening motivational determination among people experiencing stress related to various dimensions of their lives to reduce the risk of burnout. Another possible explanation for the discrepant result may be due to other tools used in earlier studies. Based on research, we often observe that different measures can yield different findings as they may assess distinctive aspects of the construct under study.

Our findings should be viewed in the light of certain limitations. The current study has a cross-sectional design that does not allow any causal relationships to be deduced between the variables considered. However, there is theoretical justification for the choice of the predictors used in the current study. In addition, while the measurement of selected confounders was an important element of our study, expanding the pool of other potential confounders (e.g., the type of employment, economic conditions) could bring new insight into understanding the mediation relationship between stress and burnout. Moreover, the data were collected using self-report measures that might increase desirability bias and affect the validity of the results. Accordingly, a careful interpretation is recommended when applying the results.

## Data Availability

All data have been made publicly available at osf and can be accessed at https://osf.io/ax3jf/?view_only=1b4c1e3d82324844a202f1be56804f6d.
